# The Standard of Work on the Natural Teeth That the Dentist of the Future Must Be Equal to

**Published:** 1885-02

**Authors:** E. Parmly Brown

**Affiliations:** Flushing, N. Y.


### THE STANDARD OF WORK ON THE NATURAL TEETH THAT THE DENTIST OF THE FUTURE MUST BE EQUAL TO.

BY E. PARMLY BROWN, D. D. S., FLUSHING, N. Y.

The highest type of tooth preparation for gold building on decayed teeth is that which removes the most of the damaged tooth material, and cuts into and takes away the least of the uninjured structure, all other things being equal.

The most durable gold filling, applied to this ideal cavity, is that which is most dense up to a certain requirement, from the begining of the retaining pits to the final finished surface. Density, combined with cohesion in its most perfect state, is requisite at each under-cut, retaining pit or dove-tail, to retain the filling in position, and to enable the operation to progress from its beginning to its completion without its budging, and finally to permit its being used for years or a life-time without giving way. The density of the center should be sufficient to prevent interference with permanent strength, or with the ability of the operator to accomplish all the auxiliary necessities. The density of the surface must be sufficient to permit the smooth and spotless finishing, and the permanent retention of artistic contour. The density of the margins of gold, where the tooth structure and filling join, must be sufficient to prevent and preserve a smooth and unbroken edge.

The highest type of enamel or tooth margin, which is to meet the gold margin, is that which is the farthest from forming a sharp edge or acute angle, to avoid the crumbling of the margin during insertion of the gold, as well as to prevent its breaking down by usage and time.

The highest type of gold margin which is to join the tooth margin is that which leaves the gold the farthest from a feather edge. And as these margins of different substances are always to meet each other, an obtuse angle to its edge must be given that which needs it the most, the enamel, which is of a brittle nature ; the one that should receive the most acute angle to its edge will be that which will bear it the best, viz., the gold, which is malleable, lead-like.

The highest type of completed filling is that which is truest to nature; that which will most minister to usefulness and comfort, and best prevent future decay. This means a thorough contour restoration, illustrated on the grinding surfaces of the teeth by close occlusion of the filling with its antagonizing tooth; illustrated on the approximal surfaces by close knuckling of the gold to its next door neighbor to prevent the crowding between the teeth of food, which destroys the inter-dental portion of the festoon of the gum, causing pain and inviting decay. The close knuckling also prevents an approaching movement of the teeth, which without the knuckling would in many cases meet each other at the margin of the gum, destroy it and invite decay by contact at that point. The accident most apt to befall this class of work is the entire breaking down of the gold, when improperly inserted, an accident preferable to the insidious lurking of decay behind the other class of fillings, imperfectly and loosely packed within weak retaining walls, when disastrous inroads of destruction warn the patient when it is too late, in most cases, to save the pulp or tooth.

The highest type of the dental arch completely repaired, durability and comfort being taken for granted, is that which is the truest to nature that the circumstances of the case will permit.

The highest type of gold is that which will do this work the best, the ease to the patient and operator being considered. An easy working cohesive foil is the desirable material.

The highest type of appliances to produce the result desired is that which will accomplish it the best, in the least time, with the least pain to the patient, and the least wear and tear and most profit to the operator. The hand mallet is good, but has two great objections that a mallet or plugger propelled by other force has not; it necessitates an assistant to use it, and it is slower in delivering blows. A plugger propelled by foot power does not give the operator the range of free action that one does that is operated by force independent of his exertions. The only attention that should be required is to start or stop the plugger at will, by a simple movement of the finger on the hand piece. A water power would be best if it were possible to get the plugger to cover the ground, but water power is something that not over one dentist in ten possesses for such use. The electro-magnetic plugger every dentist can have, the battery requiring about ten minutes' attention and ten cents expense per week, and when the battery and plugger are once comprehended and are in good working order the dentist has an assistant that cannot be equaled.

Without it the operator cannot successfully compete with the operator who has it, all other things being equal. The gold works kindly under its influence, and is made as solid and strong at all points as is required. The patient objects to it less than to any other manner of inserting gold that I have ever known. The operator is delighted and infatuated with its easy, thorough, and rapid working.

Any system of filling is defective that does not condense its gold well, anchor its gold well, and have its weak margins of tooth well trimmed away.

The Herbst rotary method of inserting gold permits only one of these things. It does what the old hand pressure did, only in a different way. Its fillings are well anchored within the weak walls of the margins of the decay. The Herbst method necessitates a matrix in many or most approximal fillings, and this is a great objection because of the complication, the matrix being a very difficult thing to apply to the infinite variety of cases met with in practice.

That Herbst performs these operations skillfully and far better than the average of the dentists practicing about him, I do not doubt; but the attempts to demonstrate the superiority of the practice in this country have been lamentable failures. The rotary method of placing gold in teeth, I am happy to say as an American dentist, never would have had its birth in this land of ours, where dentistry was lifted from the gutter by our fathers, and where to-day it holds a pre-eminent position. I am willing to meet the inventor of the rotary method of packing gold, in a friendly trial of the comparative merits of his *modus operandi* with the plan of operation laid down as the correct thing in this paper. I will meet him in New York or Europe. I will operate with him in the mouth of a dentist who needs several days' work performed, he also to work in such a mouth. We will work together for several consecutive days before an audience of dentists, and let the audience see and believe, as their better judgment dictates; and if the gold building principles of Atkinson, Varney and Webb go to the wall, I am perfectly willing to go there with them, and Herbst shall triumph.

This adverse criticism of the Herbst method I make fearlessly, and I am willing to abide by the verdict that time will render. It will put many dentists struggling to attain good means to proper ends on their guard. There are many men lacking ability and knowledge to do as well as they wish, who are ever ready to grasp at every straw they see floating on the surface, instead of striking out manfully for something that will carry them safely to the harbor of success.

